# Renal Side Effects of Non-Steroidal Anti-Inflammatory Drugs in Neonates

**DOI:** 10.3390/ph3020393

**Published:** 2014-04-03

**Authors:** Karel Allegaert, Jan de Hoon, Anne Debeer, Marc Gewillig

**Affiliations:** 1 Neonatal Intensive Care Unit, University Hospitals Leuven, Herestraat 49, 3000 Leuven, Belgium; E-Mail: anne.debeer@uz.kuleuven.ac.be (A.D.); 2 Center for Clinical Pharmacology, University Hospitals Leuven, Herestraat 49, 3000 Leuven, Belgium; E-Mail: jan.dehoon@uz.kuleuven.ac.be (J.d.H.); 3 Department of Pediatric Cardiology, University Hospitals Leuven, Herestraat 49, 3000 Leuven, Belgium; E-Mail: marc.gewillig@uz.kuleuven.ac.be (M.G.)

**Keywords:** non-steroidal anti-inflammatory drugs, renal side effects, quantification

## Abstract

Non-steroidal anti-inflammatory drugs like ibuprofen or indomethacin are commonly prescribed drugs to induce pharmacologic closure of a patent ductus arteriosus in preterm neonates. Based on a recently published Cochrane meta-analysis, both drugs are equally effective to induce closure. Drug choice can therefore be based on differences in side effects or pharmaco-economic arguments. The current review quantifies the negative impact of either ibuprofen or indomethacin on renal function, including diuresis, glomerular filtration rate and renal tubular function. Both ibuprofen and indomethacin have a quantifiable impact on renal function. However, compared to ibuprofen, the negative impact of indomethacin is more pronounced.

## 1. Introduction

Renal function includes diuresis, glomerular filtration (GFR) and renal tubular activity (resorption/secretion). All these processes display maturational changes throughout childhood, but these changes are most prominent in early life. A thorough understanding of developmental changes in neonatal renal functions is needed to estimate the renal clearance capacity and the daily needs to maintain the water and electrolytes balance within physiologic ranges. Similarly, renal clearance capacity will be of relevance when elimination clearance characteristics for exogenous compounds, like drugs are considered. At birth, anatomic and functional renal immaturity limits both glomerular and tubular functional capacity with subsequent rapid increase with increasing postmenstrual and/or postnatal age. The main factors involved in renal function at birth are gestational age and the impressive renal and extra-renal hemodynamic changes (e.g., increased cardiac output, increased renal blood flow) resulting in a higher filtration rate at the glomerular membrane. Similarly, tubular transport activities mature with increasing age [1,2,3,4].

Glomerular filtration rate in neonates is very low (2–4 mL/min or 20 mL/min/1.73 m^2^) and can only be maintained due to a delicate balance between vasodilatory effects at the afferent and vasoconstrictor effects at the efferent glomerular arterioli [1,2,3,4]. These forces recruit maximal transmembrane filtration pressure in the glomerulus in a setting of overall low mean arterial blood pressure (30–40 mmHg) and high intrarenal vascular resistance. This delicate balance however results in relative limited capacity to adapt to endogenous (e.g., peripartal asphyxia, hypotension, sepsis) or exogenous (e.g., nephrotoxic drugs like aminoglycosides or non-steroidal anti-inflammatory drugs) stressors in early life [1,2,3,4].

Renal dysfunction in preterm newborns often results from the combined effect of prerenal factors that may reduce renal perfusion and/or oxygenation, and prematurity itself, increasing the risk of an acute renal failure during the first weeks. Moreover, respiratory distress syndrome (RDS) that needs mechanical ventilation with a high mean airway pressure and/or continuous positive airway pressure may also exert deleterious effects on renal hemodynamics. In fact, any other pathological increase in vasoconstriction during the neonatal period, such as metabolic acidosis, asphyxia and thermic dysregulation, may slow down the maturation process and reduce renal perfusion. Finally, polymorphisms (e.g., renin-angiotensin-aldosterone axis) may further contribute to the interindividual variability in perinatal renal function [1,2,3,4].

To illustrate the multifactorial character of perinatal renal function and the covariates of its impairment, we would like to refer to a recently published Italian multicenter study on postnatal renal function in a representative population sample of 246 preterm newborns [5]. Compared to control preterm cases, preterms with impaired renal function displayed a marked increase in creatinine values from the 3rd day of life, with still significant differences on days 7 and 10. Clinical characteristics of these indices patients were compared with the clinical characteristics in control preterm newborns admitted in the same unit. Whereas many risk factors correlated (univariate analysis) with impaired renal function, the multivariate analysis identified only five risk factors as independent covariates: maternal consumption of non-steroidal anti-inflammatory drugs (*i.e.*, indomethacin as a tocolytic) during pregnancy [odds ratio: 7.38, 95% confidence interval 3.26–16.7] and intubation at birth (odds ratio: 4.39, 95% confidence interval: 1.2–16.3) were the main risk factors. Respiratory distress syndrome, a low Apgar score and postnatal ibuprofen treatment to induce closure of a patent ductus arteriosus were identified as additional independent risk factors [5]. These epidemiologic observations are in line with other cohorts reported in literature and confirm the multifactorial origin of acute renal impairment in newborns [6,7,8,9].

It is hereby of relevance that pharmacological treatment with non-steroidal anti-inflammatory drugs either before or after birth negatively influence neonatal renal function. However, in addition to very rare and specific renal diseases (e.g., Barrter or Gitelman syndrome), non-steroidal anti-inflammatory drugs like ibuprofen or indomethacin are frequently administered in early life to induce pharmacologic constriction of a patent (symptomatic) ductus arteriosus [10]. The current review focuses on the renal side-effects of non-steroidal anti-inflammatory drugs following administration to preterm neonates to induce pharmacologic closure of a patent ductus arteriosus.

## 2. Patent Ductus Arteriosus in Preterm Neonates [10]

### 2.1. The ductus arteriosus in perinatal life

In *fetal life*, the ductus arteriosus allows most (>85%) of the combined left and right ventricular output to be diverted away from the pulmonary circulation to the low resistance fetal systemic and subsequent placental circulation. Pulmonary arterial resistance is high and pulmonary arterial perfusion is below 15% of the fetal cardiac output. Patency of the ductus arteriosus is therefore essential and crucial for fetal survival. This patency is maintained in a setting of overall low oxygen concentrations, high blood flow conditions through the ductus as well as high prostaglandin concentrations of mainly placental origin. The ductus arteriosus is an important structure during fetal life as it allows unloading of the right ventricle and joins the pulmonary trunk to the aorta with a diameter equivalent to these two major vessels. The fetal right ventricle ejects 60–65% of the combined cardiac output, of which 90% is shunted via the ductus to the aorta. The physiology and structure of the ductus arteriosus differs considerably from the two adjacent vessels, and strongly depends on the presence of prostaglandins in the fetal circulation [10].

Intra-uterine dysfunction of the ductus arteriosus is an acknowledged event, but seems to be rare. The majority of cases are probably subclinical or mildly symptomatic and therefore not diagnosed. In only few cases, ductal dysfunctions will come to the attention of the fetologist, neonatologist, or pediatric cardiologist [10,11]. Since literature regarding this entity is scant and essentially consists of anecdotal case reports, our group performed a retrospective analysis of fetal (n = 602) and neonatal cardiac ultrasound databases (n = 1,477) between 1998 and 2007. Clinical and imaging studies (*i.e.*, ultrasound) were reviewed for pathology due to or associated with premature closure of the duct. Twelve cases were identified. Eight (1.3%) were diagnosed prenatally at a median gestational age of 29 weeks (range: 20.0–37.5 weeks). The most common features on ultrasound were: excessive right ventricular (RV) hypertrophy (100%), more than expected tricuspid and pulmonary regurgitation (100% and 92%, respectively), and right atrial dilation (75%). Premature induction of delivery was advised for five patients. Neonatal therapy consisted of observation and oxygen administration (n = 7), ventilation with pulmonary vasodilators (n = 5), and one neonate required extracorporeal membrane oxygenation. There were three deaths due to respiratory failure with severe pulmonary hypertension.During follow-up, two children required additional right heart procedures and one developed a non-compaction cardiomyopathy [11].

This retrospective study illustrated at least the presence of and the clinical relevance of inadverted premature closure of the fetal ductus arteriosus. The most frequent claimed ‘cause’ for fetal premature closure of the ductus arteriosus is spontaneous idiopathic closure. However, taking the focus of this special issue on non-steroidal anti-inflammatory drugs into account, it is important to draw the attention on the fact that non-steroidal anti-inflammatory drugs like indomethacin are known to cause ductal constriction if ingested late in pregnancy. This might have been the case in at least 2/12 of our patients. One additional mother took indomethacin at 20 weeks of gestation, an age where most literature would state that there is insufficient smooth muscle for the duct to constrict.

In *(near)term neonates*, the ductus arteriosus usually closes within the first day(s) of postnatal life, starting with a functional closure (constriction) and followed by subsequent vascular remodeling. The ductus arteriosus is programmed to constrict shortly after birth, based on maturational changes in the tunica media and intima already initiated before birth. Secondary anatomic remodeling and permanent closure of the ductus require profound hypoxia within the constricted vessel wall. Hypoxia in the ductal vasa vasorum induces angiogenesis, neointima formation and apoptosis. VEGF (vascular endothelial cell growth factor), fibronectin, laminin, integrins and endothelin-1 are involved in this remodeling process. Ductus arteriosus smooth muscle cell migration is dependent on laminin and its receptors. Together, all these processes finally result in anatomic closure of the ductus [10,12].

Contributors to this normal physiological adaptation are the significant increase in arterial oxygen concentration after delivery, the decrease in serum prostaglandins after disconnection for the placental circulation, and the decrease blood flow through the ductus arteriosus due to the decrease in pulmonary vascular resistance. Because persistence of a patent ductus arteriosus in (near)term neonates is rare compared to preterm neonates, clinicians should consider either anatomic malformations (e.g., aneurysma) or hypothyroidism, since thyroid hormone intervenes in several key pathways involved in functional and anatomic closure of the ductus [12].

In *preterm infants*, multiple epidemiologic studies throughout the world documented that spontaneous postnatal closure of the ductus arteriosus is delayed or even does not occur [10]. The persistence of the patent ductus arteriosus in preterm infants is inversely related to gestational age and birth weight. The incidence of PDA is 70% in preterm infants weighing less than 1,000 g and 29 weeks gestational age. Although spontaneous closure of the ductus will finally occur in approximately 34% of these extremely low-birth-weight (ELBW, *i.e.*, < 1,000 g) infants, failure of the ductus arteriosus to close in the remaining infants can result in potential morbidity or mortality [13].

This is due to the fact that failure of the patent ductus arteriosus to close results in hemodynamically significant left to right shunting of blood and undesirable pulmonary, renal and gastrointestinal effects, including pulmonary edema and hemorrhage, congestive cardiac failure, intraventricular hemorrhage, cerebral vascular accidents, necrotizing enterocolitis, feeding intolerance, poor weight gain, bronchopulmonary dysplasia and/or death. Therefore, diagnosis and appropriate treatment of a patent ductus arteriosus is essential to prevent these morbidities [10,13].

In conclusion, the patent ductus arteriosus is a crucial connection between systemic and pulmonary arterial circulation of relevance throughout perinatal life. Although there are reports on preterm fetal closure as well as persistent patency in term neonates, the most relevant population to consider is the preterm neonate in early neonatal life.

### 2.2. Treatment of a symptomatic patent ductus arteriosus in preterm neonates

As mentioned earlier, a symptomatic patent ductus arteriosus may result in important haemodynamic changes causing respiratory, gastro-intestinal, intracerebral lesions and/or renal failure. Symptomatic patent ductus arteriosus presents with bounding pulses with wide pulse pressure, hyperdynamic precardium, heart murmur, worsening respiratory status, (lung)edema and/or oliguria. Since these clinical symptoms are neither sensitive nor specific enough, echocardiogram remains the gold standard to confirm the presence and the significance of a patent ductus arteriosus [10,11,13]. Echocardiographic criteria to quantify the clinical significance of a patent ductus arteriosus are the presence of a ductal size of > 1.5 mm, a ratio of the left atrium/aorta > 1, the presence of left-to-right shunting of blood, end diastolic reversal of blood flow in the aorta and/or poor cardiac function. Once the diagnosis has been made, currently reported therapeutic options to consider are *either* a conservative-supportive approach, *or* surgical closure *or* pharmacologic treatment with one of the non-steroidal anti-inflammatory drugs (*i.e.*, ibuprofen or indomethacin). These different options in part also reflect the existing controversy in literature on the management of patent ductus arteriosus in preterm neonates [13,14].

Conservative medical management involves fluid restriction, watchful waiting and ventilator support. This approach is associated with a high failure rate, especially in low-birth-weight infants and at least results in prolonged ventilatory and prolonged intensive care in combination with inadequate caloric intake due to the fluid restriction [11,13,15]. Based on the currently available evidence on the need to induce anabolic metabolism as soon as feasible after preterm delivery, it seems that conservative, expectative management is suboptimal although there are no randomized controlled trials comparing expectative versus curative (either medical or surgical) management of a symptomatic patent ductus arteriosus. In contrast, a primary surgical approach with ductal ligation seems too aggressive, moreover since recent studies at least suggested an association between the need for surgical ligation and subsequent neuorsensory impairment and neurodevelopmental delay [16]. It is however important to mention that surgery was only performed in cases in whom initial medical treatment with non-steroidal anti-inflammatory drugs had failed. Pharmacological treatment with one of the two currently available non-steroidal anti-inflammatory drugs seems the most balanced approach and is perceived to the therapy of choice for safe and effective treatment of a symptomatic patent ductus arteriosus [14].

Pharmacological treatment with one of the two currently registered drugs (ibuprofen or indomethacin) results in closure of the patent ductus arteriosus in 70 to 80% of the extreme low birth weight infants, while the use of aspirin became obsolete because of the lower closure rate as compared to indomethacin [10]. The optimal time to treat a patent ductus arteriosus is when treatment with a non-steroidal anti-inflammatory drug will be most effective while avoiding treatment of infants in whom the patent ductus arteriosus may still evolve to spontaneous closure. Prophylaxis in very early life to induce closure of a patent ductus arteriosus and to improve subsequent outcome (neurodevelopmental, respiratory) has been extensively evaluated and turned out to be ineffective for both indomethacin (despite the reduction in incidence in intraventricular hemorrhages) and ibuprofen (despite the reduction in bronchopulmonary dysplasia in the initial meta-analysis, more recently no longer present) [17,18]. The current trend seems to be to treat early presymptomatic patent ductus arteriosus at 2 to 7 days of postnatal life after confirmation by cardiac ultrasound. Such an approach will limit the total number of infants exposed to non-steroidal anti-inflammatory drugs whose ductus will close spontaneously while still be beneficial for those cases who will respond to medical induced closure.

Pharmacological treatment with one of the two currently registered drugs (ibuprofen or indomethacin) results in closure of the patent ductus arteriosus in 70 to 80% of the extreme low birth weight infants and there is no difference in effectiveness between both drugs [10,13,14]. Meta-analysis of studies comparing ibuprofen to indomethacin showed no significant differences in the incidence of necrotizing enterocolitis, bronchopulmonary dysplasia at 36 weeks postmenstrual age nor neurodevelopmental outcome at 18 months of age [19]. It is however well known that these drugs also cause renal (side) effects since renal perfusion and diuresis in early life strongly depend on the vasodilative effects of prostaglandins on the afferent glomerular arterioli. Both non-selective non-steroidal anti-inflammatory drugs (e.g., aspirin, ibuprofen or indomethacin) as well as more selective cyclooxygenase-2 inhibitors (e.g., rofecoxib) abolish the vasodilatory effects of prostaglandins, resulting in reversible vasoconstrictive renal hypoperfusion [20,21]. In the absence of any significant difference in effectiveness of ductal closure rate between both drugs, drug choice can be made based on differences in side effects or pharmaco-economy arguments. In the current review, we aimed to quantify the negative impact of either ibuprofen or indomethacin on renal function, including diuresis, glomerular filtration rate and renal tubular function in early neonatal life.

## 3. Quantification of renal impairment: ibuprofen versus indomethacin

### 3.1. Diuresis

Due to the presence of placebo-arms in the placebo-controlled prophylactic trials that evaluated the potential impact of early prophylactic administration of either indomethacin or ibuprofen, these trials also provided clinicians with important additional information on the normal physiologic adaptations of perinatal renal function, including diuresis in the first days after preterm birth. In the placebo arm of these studies, diuresis displays a postnatal progressive increase (from 2.3, SD 1.4 on day 1 to 4, SD 1.6 mL/kg/h on day 3) [17,18]. Secondly, these placebo-controlled trials clearly documented that both ibuprofen as well as indomethacin affect diuresis. In the indomethacin propylaxis study, incidence of oliguria doubled in the indomethacin treated group (44/601 *versus* 22/601), while ibuprofen administration as prophylaxis in early neonatal life results in a initial decrease in diuresis (2.3, SD 1.4 to 1.1 (SD 1.1) mL/kg/h) on day 1 and an increase in the incidence of oliguria (30/210 to 45/205). Based on the currently available compounds, there is no such a thing as a non-steroidal anti-inflammatory drug without renal side effects [17,18].

The compound-to-compound evaluation is obviously of more clinical relevance when drug choice is considered. A meta-analysis of studies comparing the efficacy of either indomethacin or ibuprofen on PDA closure also focused on renal function and hereby clearly documented that the reduction in diuresis is more pronounced in neonates co-treated with indomethacin compared to neonates treated with ibuprofen. This is reflected in the difference in incidence of oliguria (< 0.5 mL/kg/h) and in differences of median diuresis. Van Overmeire *et al.* documented oliguria in 5/74 during ibuprofen administration and in 14/74 during indomethacin administration (p < 0.05) [22]. When median daily diuresis in the first week of postnatal life was evaluated, it was documented that diuresis from day 3 to day 7 was more impaired following indomethacin administration (reduction of 1 to 1.5 mL/kg/h, equal to 25–40% in daily urine production). Median urine output was 3.1 *vs.* 3.8 mL/kg/h on day 3, with a subsequent decrease in both groups to 2.1 *vs.* 3 mL/kg/h on day 5. Based on the available study cohorts, the number-needed-to-harm (NNH, avoidance of oliguria) has been calculated to be 8 in favor of ibuprofen [10,19,22].

These differences in effects of indomethacin and ibuprofen on diuresis are likely explained by the different effects of both drugs on renal blood flow in preterm neonates. Based on renal blood flow Doppler observations in 17 preterm neonates, it was concluded that renal blood flow velocity decreased significantly and did not return to pretreatment values in the first 120 min after indomethacin administration, while this reduction of renal blood flow was not observed after ibuprofen administration [20,21].

### 3.2. Impairment of glomerular filtration capacity: creatinaemia or renal drug clearance

The above already mentioned placebo-controlled studies also provided us with additional information on normal postnatal trends in creatinaemia, and hereby confirmed other studies on covariates of perinatal variability in creatinaemia. Akima *et al.* documented that indomethacin administration results in a significant increase (+25%) of creatinaemia in 24% of preterm neonates [23]. Creatinaemia remained elevated at least up to one week after administration of indomethacin. Creatinine clearance decreased up to at least day 7 (23.6, SD 9.8 *vs*. 27.6, SD 8 mL/kg/1.73m^2^) in indomethacin treated cases (relative decrease 15%). Very recently, Vieux *et al.* quantified the negative impact of ibuprofen administration on creatinine clearance in extreme preterms in a case-control setting (symptomatic patent ductus arteriosus needing ibuprofen *vs.* control patients without patent ductus arteriosus) [24]. Creatinine clearance on day 7 was 12.8, SD 6.2 *vs.* 18.1, SD 12.1 mL/min/1.73m^2^) in ibuprofen treated cases (relative decrease of 30%). Unfortunately, the clinical characteristics of both cohorts are different, as also reflected in the differences in creatinaemia clearance between both control groups, making compound-to-compound comparison hazardous. At least, it reconfirms that both indomethacin and ibuprofen have an impact of creatinine clearance.

Compound-to-compound evaluations in preterms with symptomatic patent ductus arteriosus and with specific emphasis on creatinaemia have been published. Creatinaemia was significantly higher from day 4 to day 8 in preterms treated with indomethacin compared to ibuprofen. In the most recent meta-analysis, observations on creatinaemia in 386 preterms, creatinaemia on day 3 after administration was significantly lower (- 8.2 mmol/L, equal to - 0.1 mg/dL) in the ibuprofen treated group [25].

This Cochrane meta-analysis is further supported by epidemiologic observations as reported by, among others, Fanos and coworkers [26]. Using a retrospective analysis of clinical data in a single neonatal intensive care unit, renal tolerability of ibuprofen and indomethacin administered to preterm infants with gestational age (GA) < 30 weeks was evaluated. Once again, these authors confirmed that both ibuprofen and indomethacin transiently affect renal function, but creatinine concentrations normalized slower in indomethacin treated cases compared to ibuprofen [26]. It is however also important to be aware that the use of creatinaemia values to quantify creatinine clearance and/or glomerular filtration rate in preterm neonates is in part also hampered because of specific methodologic aspects and perinatal renal adaptive processes [3,27].

Firstly, it is important to consider the biochemical assay used to quantify creatinaemia in neonatal samples. The historically used ‘Jaffe’ technique is a colorimetric technique but interference with cephalosporins, bilirubin and ketoacids has been described. Cephalosporins are routinely prescribed in neonates while hyperbilirubinemia is a frequent finding in the first week of postnatal life. Enzymatic quantification seems to be more appropriated [3].

Secondly, one should be aware that creatinaemia at birth still mainly reflects maternal renal function and only becomes a more reliable marker of glomerular filtration after the first days of postnatal life [27]. In term neonates, serum creatinaemia decreases rapidly to reach neonatal levels of 0.4 mg/dL by 1 or 2 weeks of postnatal age, while in very preterm neonates there is a transient increase in creatinaemia with a peak on day 3–4 of postnatal life with a subsequent progressive decrease over the next 3 to 4 weeks. This transient increase is a least in part due to passive renal tubular ‘back-leak’ across renal tubular cells lines, making creatinaemia an unreliable marker of glomerular filtration in early life while in adults there is a small net secretion of creatinine so that creatinine clearance is very close to glomerular filtration rate [6,7,8,27].

One of the available surrogate markers to quantify the impact of renal side effects of non-steroidal anti-inflammatory drugs in neonates, is to use the impact of these compounds on elimination clearance of other drugs that strongly dependent on glomerular filtration for elimination. Strong correlations between aminoglycoside and creatinine clearance are reported in human neonates while glycopeptides like vancomycin are also almost exclusively cleared by renal elimination [28].

Both drugs are frequently administered in neonates and data from therapeutic drug monitoring are available, making such an approach both feasible and of clinical relevance. The implementation of population pharmacokinetics tools using non-linear mixed effects models enabled us to disentangle the impact of different covariates, including co-administration of non-steroidal anti-inflammatory drugs on aminoglycosides (amikacin) and vancomcyin clearance in neonates [4,9,29,30,31,32].

In consecutive efforts, we quantified covariates of amikacin clearance in preterm neonates, including the impact of ibuprofen co-administration [29,31,33]. In cases co-treated with ibuprofen, amikacin clearance was 21% lower compared to cases not co-treated with ibuprofen, independent of the gestational age at birth [33]. To further estimate the impact of symptomatic versus prophylactic administration of ibuprofen, amikacin pharmacokinetics were estimated in a further extended cohort of 715 neonates (PMA 24–43 weeks, weight 0.385 to 4.78 kg), based on 1 862 amikacin time-concentration profiles of which 5% were co-treated with ibuprofen. The impact of ibuprofen co-administration on amikacin clearance was of similar magnitude (20–25%), independent of the clinical indication (prophylaxic versus therapeutic administration) [29].

To further confirm the magnitude (clearance reduction of 20 to 25%) of this effect, we quantified the impact of ibuprofen co-administration on vancomycin elimination in the first month of life. Based on data from 604 vancomycin time-concentration profiles in 214 subjects entered in a population pharmacokinetic study, size/weight (50%), age (18%), renal function (creatinaemia) (14%) explained 82% of the interindividual variability in vancomycin clearance observed. Co-administration of vancomycin (17/214 cases) resulted in 18% reduction in vancomycin clearance [32].

Finally, and in an effort to compare the impact of ibuprofen versus indomethacin on renal drug clearance capacity, a pooled pharmacokinetic study was undertaken to compare the effects of co-administration of either ibuprofen or indomethacin on renal clearance of vancomycin in preterm neonates. Based on a dataset of 883 time-concentration profiles in 365 neonates (median 1.3 kg, range 0.58 to 3 kg) of which 7% were co-treated with ibuprofen and 9% with indomethacin, indomethacin and ibuprofen reduced vancomycin renal clearance by 46% and 28% respectively [34].

The observations on the differences in vancomycin clearance after indomethacin or ibuprofen administration described in this pooled analysis strongly suggest that ibuprofen has less of a deleterious effect on renal drug clearance in preterm neonates [34]. The difference in drug clearance reduction in support of ibuprofen is in line with the differences in urine flow and serum creatinine previously described (3.1 and 3.2).

### 3.3. Renal tubular functions

A comprehensive description of the renal effects of these drugs should include variables such as urine production, glomerular filtration and tubular secretion/resorption of important physiological substances and exogenous compounds. Knowledge on the ontogeny of renal tubular cell transport processes is still very limited [3]. In the absence of knowledge of these processes, one can only speculate on the impact of either ibuprofen or indomethacin and the differences between both compounds on the phenotypic renal tubular activity in early life. Hyponatriaemia will hereby only very partially reflect renal tubular dysfunction since hyponatriaemia can also be a marker of fluid retention.

To the very best of our knowledge, data on the impact of non-steroidal anti-inflammatory drugs on renal tubular functions are only available—to a certain extent—after ibuprofen administration. Vieux *et al.* reported on the impairment of renal tubular function up to 3 weeks after administration of ibuprofen [24]. Indicators of renal tubular function were fractional sodium excretion and quantification of urinary microalbumin/creatinuria and alpha-1-microglobulin/creatinuria. Median fractional sodium excretion was significantly higher on day 7 (3.9 *vs.* 2.6%), 14 (2.3 *vs.* 1.4%) and 21 (1.6 *vs.* 1.1%) in ibuprofen treated neonates. The ratio microalbuminuria/creatininuria was also significantly higher up to day 14 in ibuprofen treated cases.

Similar observations on the impact of indomethacin are not available, so we can only speculate on the extent of its effect. Since the effects on renal tubular function relate to the extent of preglomerular arteriole vasoconstriction, it is to be anticipated that indomethacin will have at least similar negative effect on renal tubular functions.

## 4. General Discussion and Conclusions

Treatment options currently available for a symptomatic patent ductus are either the use of a conservative approach, or primary surgical ligation, or the administration of a non-steroidal anti-inflammatory drug. Pharmacological treatment with one of the two currently available non-steroidal anti-inflammatory drugs seems the most balanced approach and is perceived to the therapy of choice for safe and effective treatment of a symptomatic patent ductus arteriosus. Since ibuprofen and indomethacin are equally effective in closing a patent ductus arteriosus, it is of relevance to generate data on the safety profile of non-steroidal anti-inflammatory drugs in neonates [25]. This should enable the clinician to make a balanced decision, based on perceived benefits and risks in the individual patient.

In this review, we focused on the renal side effects of both drugs. Based on the available evidence, we hereby confirmed that the administration of ibuprofen or indomethacin both result in transient renal impairment. This renal impairment is due to a decrease in pre-glomerular vasodilatation, secondary to a decrease in prostaglandin synthesis and reflects the delicate balance between vasodilatory effects at the afferent and vasoconstrictor effects at the efferent glomerular arterioli.

However, the reduction in diuresis and glomerular filtration capacity in neonates is more blunted during ibuprofen versus indomethacin administration. Compared with indomethacin, ibuprofen reduces the risk of oliguria (NNH 8) and is associated with lower serum creatinine levels following treatment while data on the differences in impairment of renal tubular functions are not yet available. As both drugs are equally effective in closing the ductus, the clinician needs to take the potential side effects into account when making a decision which drug to use. When we only focus on the renal side effects, it seems that ibuprofen is the preferred drug. In the clinical setting, these renal observations should however be balanced with other extra-renal (e.g., incidence of pulmonary hypertension, incidence of intraventricular haemorrhage) [13,25]. Irrespective of the individual choice made, the clinician has to take the renal effects into account when prescribed fluid, electrolytes or drugs cleared by renal route.

Future studies are urgently needed to document the potential impact of recently suggested dosing regimes to increase efficacy of closure since higher and prolonged exposure of ibuprofen might resulted in more pronounced, more prolonged, or even more persistent renal impairment [35]. Similarly, adaptations in either the route of administration (oral versus intravenous) or the use of enantiomer specific solutions have to be evaluated on their potential benefits compared to the currently implemented dosing regimes [36,37]. Others reported on the improved renal tolerance to indomethacin when continuous administration was compared to intermittent ‘bolus’ administration [38]. Finally, data on long term renal outcome following perinatal exposure of non-steroidal anti-inflammatory drugs are urgently needed to guide future clinical care. The patients initially recruited in the randomized-controlled trials mentioned earlier might hereby generate the strongest scientific evidence on long term safety issues.
